# Clomiphene citrate impairs the endometrial CD98 expression in ovariectomized and non-ovariectomized rats: Role of HCG

**DOI:** 10.18502/ijrm.v17i6.4809

**Published:** 2019-07-29

**Authors:** Behpour Yousefi, Elnaz Rahbar

**Affiliations:** ^1^Abnormal Uterine Bleeding Research Center, Semnan University of Medical Sciences, Semnan, Iran.; ^2^Department of Anatomy, Medicine Faculty, Semnan University of Medical Science, Semnan, Iran.

**Keywords:** Endometrium, Clomiphene citrate, Human chorionic gonadotropin, CD98.

## Abstract

**Background:**

Clomiphene citrate (CC) is one of the widely used drugs as an ovulation stimulant, but its adverse effects on the endometrium results in lowering down the pregnancy rate. Endometrium CD98 is also important in the process of implantation.

**Objective:**

To evaluate the immunohistochemistry expression levels of endometrial CD98 following injection of CC with and without Human chorionic gonadotropin (HCG) in ovariectomized and non-ovariectomized rats.

**Materials and Methods:**

Seventy two (12-14 wk old) female Wistar rats were randomly divided into two groups (n = 36): (a) ovariectomized and (b) non-ovariectomized. Each group was further divided into six subgroups (n = 6/each): (1) CC 10 mg/kg, (2) CC 20 mg/kg, (3) HCG, (4) CC 10 mg/kg with HCG, (5) CC 20 mg/kg with HCG, and (6) control. The experimental subgroups received a single dose of CC (daily, five days) and HCG (after the last injection of CC) alone or in combination. Immunohistochemistry staining was performed on paraffin-embedded endometrial tissues to evaluate the expression levels of CD98.

**Results:**

Animals undergoing ovariectomy presented a significantly lower expression level of endometrial CD98 (p < 0.001) when compared with non-ovariectomized in the same condition that groups were subdivided. There was also a dose-dependent reduction (p < 0.001) in the expression of CD98 in non-ovariectomized subgroups when compared with control group. In addition, injection of HCG following treatment with CC improved its expression.

**Conclusion:**

It was concluded that CC impairs CD98 expression in endometrium and this impairment is intensified *with* the removal of the ovary. Also, an injection of HCG following treatment with CC can slightly improve the expression of CD98.

## 1. Introduction

Clomiphene citrate (CC) is advised to be the first-line treatment for polycystic ovary syndrome (PCOS) (1) and other subfertility illnesses associated with oligo-ovulation (2) since it is effective, low-cost, easily available, and the majority of women tolerate it well (1); it is increase the ovulation rate (60-85%) but decrease the pregnancy rate (only by about 20%) due to its adverse effects on the endometrium (2). In addition, histological and biochemical studies confirm these detrimental effects of CC (3, 4). Several studies have revealed that CC, depending on the endogenous hormonal environment can be an estrogen agonist either an antagonist on estrogen-sensitive tissues (5) so that the public use of the intrauterine insemination (IUI) almost is owing anti-oestrogenic effects of CC on the cervical mucus (4).

Also, according to the previous studies, the administration of HCG following pre-stimulation with CC inhibits detrimental effects of CC on the endometrial competence and therefore increases the development, proliferation, and thickness of endometrium (6).

A biochemical, immunohistological, and functional study has revealed that CD98 is a key factor of human endometrial receptivity, as its expression is strongly regulated during the implantation window (7, 8). Implantation window is a specific period of the uterine cycle when the endometrium is highly receptive for blastocyst (9) and takes place for 4-5 days after progesterone secretion and lasts about five days. It is also framed by the co-expression of three members of the integrin family, α1β1, α4β1, and αvβ3 (10) and steroidal ovarian hormones (11). At this stage, the endometrium undergoes morphological [modifications of the plasma membrane (12) and cytoskeleton (13)], structural and biochemical changes under the influence of specific gene regulation. CD98/4F2/FRP-1(fusion regulatory protein-1) was found in human and mouse lymphocytes in 1981 and described as an activated antigen (14). It is a multifunctional protein (15) and at least two distinct functions have been descripted for its heavy chain, transportation of amino acids (16), and correlation with β1 and β3 integrins which cause an increase in their affinity for ligands and integrin signaling regulation (17, 18). CD98 has also involved in cell–cell interactions directly (19) and is necessary for cell adhesion and fusion which is essential in placenta development. (20). It is also determined that distraction of the CD98 gene increases the mortality of mouse embryos (21). All cell types express CD98 except for the platelets (22), stromal and epithelial cells of endometrium express it during the mid-secretory phase as well. It locates at the apical surface of endometrial epithelial cells and correlates with tetraspanin-enriched microdomains in a hormone-dependent organization, which relates with blastocyst adhesion and it is now clear that decreasing of expression levels of CD98 plasma membrane causes failure of blastocyst adhesion (7) and may be one of the results of recurrent implantation failure in IVF treatment receiving cases (23).

Although there are evidences of importance of CD98 in implantation and it is also obvious that hormonal changes can alter CD98 expression levels, effects of ovulation inductors like CC on endometrial CD98 expression has not been studied. The current research was planned with that need in mind.

## 2. Materials and Methods

### Animals and drug treatment

In this experimental study, 72 (12-14 wk old) virgin female Wistar rats (*Ratus norvegicus*) were obtained from the Central Animal Unit of the Iran University of Medical Sciences; CC and HCG were purchased from Sigma Chemical Company (St Louis, MO, USA) and LG Life Company (LG Life Sciences, South Korea), respectively. Solvents were analytically graded and used without further purification.

Animals were randomly divided into two main groups (n = 36/each): (I) ovariectomized and (II) non-ovariectomized. Animals were kept under standard laboratory conditions, under a 12 hr light/dark cycle at room temperature of 22 ± 2°C and had free access to pelleted food and tap water. Ovariectomized group went under bilateral ovariectomy as described by Hosie and colleagues (11) and were allowed 3-4 wk to recover before any treatments after surgery to make sure that there are no ovarian hormones left in the circulating system (9). Both ovariectomized and non-ovariectomized groups were divided into six subgroups (n = 6/each):

(a) cc10, (b) cc20, (c) cc10 + HCG, (d) cc20 + HCG, (e) HCG and control, which were kept without intervention. Animals of (a), (b), (c), and (d) subgroups received 10/20 mg/kg of CC by daily intraperitoneal injection at 10:00 am for five days. The (e) subgroup received a single dose of HCG (10 I.U. /0-4 ml 0-154 m-NaCl) at 10:00 am. The (c) and (d) subgroups both received a single dose of HCG after the last injection of CC.

### Histological study 

#### Sample preparation

All animals were sacrificed on the morning of the day after mating (a vaginal plaque and smear observed) approximately 24-26 hr after the last drug injection using a lethal dose of ketamine (150 mg/kg) and Xylasin (15 mg/kg). The left uterine horns were excised, placed in 0.1 M of phosphate buffered saline [(pH 7.4 (Gibco life tech. tablets)], cleaned of fat, cut into 1-cm pieces, and then tissues were fixed in 10% formalin solution, paraffin embedded, then cut to 5-μm thick sections (transverse serial sections); deparaffinized with xylene and rehydrated using graded ethanol.

#### Immunohistochemistry

A primary anti-CD98 antibody and an Immuno Cruz${}^{TM}$ ABC Staining Kit (Santa Cruse, USA), used in case and control groups for immunohistochemistry staining. Tissue sections were blocked in 10-mM sodium citrate buffer, pH 6.0 for 10 min at 90°C, and endogenous peroxidase activity was suppressed by incubation with 0.1% H${}_{2}$O${}_{2}$ in absolute methanol for 5 min at room temperature and a 6-min rehydration in phosphate buffered saline (PBS). After the incubation with blocking serum for 60 min at room temperature (1.5% normal goat serum), sections were incubated with the primary antibody (1:100) diluted in PBS at 4°C overnight. Negative controls were incubated without primary antibody. A PBS rinse was followed by the treatment with the secondary antibody (a biotinylated anti-rabbit IgG antibody) for 30 min. Sections then washed and incubated with avidin: biotinylated horseradish peroxidase macromolecular complex for 30 min. DAB (Diaminobanzidin) was added as the choromogene and incubated for 8 min to complete the reaction and to visualize the immunostaining. As a final step, sections sections counterstained with hematoxylin for 5 min, dehydrated in a graded series of ethanol, and cleared with xylene. A coverslip was placed over permount for evaluation by light microscopy. The endometrial blood staining used as positive internal controls as well. Images were captured at 400× magnification using a Zeiss LSM 5 light microscope evaluated by two treatment blinded observers. The assessment of staining intensity and distribution in endometrial epithelium was made using the semi-quantitative histologic score (HSCORE) system. HSCORE was calculated using the following equation: 

H score =∑Pi(I+1)/100

Where *i* = intensity of staining with a value of 1, 2, or 3, (weak, moderate, or strong, respectively) and *Pi* = the percentage of stained endometrial epithelial cells for each intensity, varying from 0-100%.

Low intra-observer (r = 0.983; p < 0.0001) and inter-observer (r = 0.994; p < 0.0001) differences for HSCORE in uterine tissues have been previously reported using this technique. The numerical cut-off for a negative result for the CD98 was an HSCORE of ≤ 0.7, based on previous ROC analysis (24).

### Ethical consideration

All animal care and experimental procedures were carried out in order to avoid or reduce pain or stress to a lab animal whenever possible and approved in the approval letter no.1862.

### Statistical analysis

Statistical calculations were performed using SPSS software (Statistical Package for Social Science, version 16.0, SPSS Inc, and Chicago, IL, USA). Statistical significance between groups was evaluated using one-way ANOVA analysis of variance. The differences between groups were determined by the Tukey test. Results were presented as mean ± SD with p < 0.05 considered to be statistically significant.

## 3. Results

### Expression of CD98 on endometrial epithelium in ovariectomized rats

Table I shows that there is no significant difference between CD98 expression levels in ovariectomized subgroups with the exception of CC10 + HCG and HCG group (p < 0.0001) which indicates that HCG can improve adverse effects of ovariectomy only when CC is not used or is used in low dosage. Table II also shows that CD98 expression levels are significantly higher in non-ovariectomized subgroups compared to ovariectomized subgroups with the same treatment which shows the importance of ovarian hormones, but when CC is used in higher dosage, the presence or lack of ovary makes no difference in the expression levels.

### Expression of CD98 on endometrial epithelium in non-ovariectomized (normal) rats

**Figure 1:**
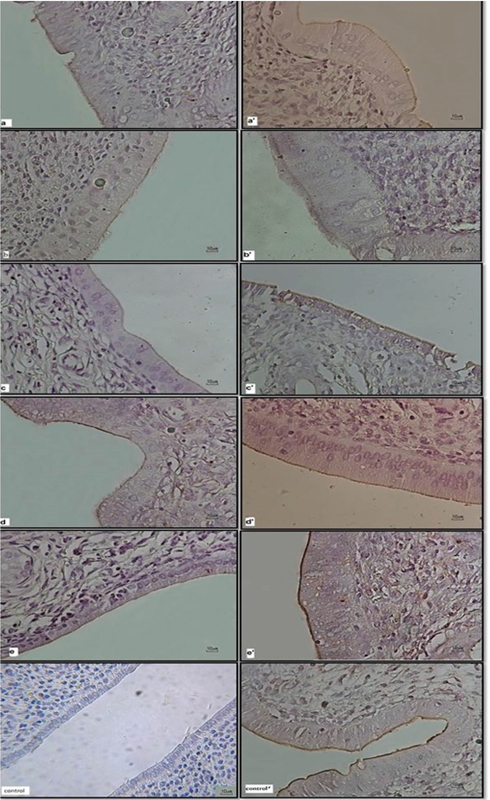
Microscopic images (10×) of rat uterus tissues in different groups one day after mating; consider the expression levels due to chromogenic severity of the apex of endothelial cells. I. Ovariectomized subgroup: (a) CC10 group; (b) CC20 group; (c) CC10 + HCG group; (d) CC20 + HCG group; (e) HCG group; (control) Control group. II. Non-ovariectomized subgroup: (a') CC10 group; (b') CC20 group; (c') CC10 + HCG group; (d') CC20 + HCG group; (e') HCG group; (control') Control group.

The comparison of the CD98 expression in the non-ovariectomized rats exposed to CC with and without HCG and HCG alone with control group is summarized in Table III. Overall there was a statistically significant decrease (p < 0.0001) in the expression of the CD98 in the non-ovariectomized rats undergoing treatment with CC (10, 20 mg/kg) alone and in combination with HCG compared to the control group. Administration of HCG following CC induced the expression, but it could not fully reverse it to normal expression status. Normal expression levels of CD98 were only observed in the subgroup treated with HCG alone (Figure 1).

### Expression of CD98 on endometrial epithelium was dependent to CC dosage

Table IV shows a significant decrease in CD98 with increasing dosage of CC from 10 to 20 mg/kg in all subgroups of non-ovariectomized group and subgroups that received HCG with CC in the ovariectomized group that shows that adverse effects of CC are dose-dependent.

### Expression of CD98 on endometrial epithelium due to HCG administration after pre-stimulation by CC

HCG significantly affects the expression of the CD98 in all subgroups when CC is used in a lower dosage, but the higher dosage of CC prevents the improvement, especially in a non-ovariectomized group (Table V).

**Table 1:** Expression levels of endometrial epithelium CD98 in ovariectomized subgroups exposed to CC with and without HCG, and comparison with control group


**Subgroups (each, n = 6)**	**Ovariectomized group**	**Control group (n = 6)**	**p** *${}^{a}$*
CC10 (mg/kg)	1.12 ± 0.30	0.99
CC20 (mg/kg)	1	0.99
HCG (7.5IU/kg)	2.24 ± 0.51	0.0001
CC10(mg/kg) + HCG (7.5I.U)	1.78 ± 0.63	0.0001
CC20(mg/kg) + HCG (7.5I.U)	1.15 ± 0.21	1.07 ± 0.71	0.99
${}^{a}$Note: a values represent mean and the associated standard deviation; p < 0.05 is considered statically significant			
One-way ANOVA and Tukey tests were used to determine significance			
CC: clomiphene citrate HCG: human chorionic gonadotropin			

**Table 2:** Expression levels of endometrial epithelium CD98 in ovariectomized and non-ovariectomized subgroups exposed to CC with and without HCG


**Subgroups(each, n = 6)**	**Ovariectomized group**	**Non-ovariectomized group**	**p** *${}^{a}$*
CC10 (mg/kg)	1.12 ± 0.30	1.86 ± 0.22	0.001
CC20 (mg/kg)	1	1.22 ± 0.28	0.972
HCG (7.5IU/kg)	2.24 ± 0.51	4	0.0001
CC10(mg/kg) + HCG (7.5I.U)	1.78 ± 0.63	2.69 ± 0.38	0.001
CC20(mg/kg) + HCG (7.5I.U)	1.15 ± 0.21	1.80 ± 0.82	0.054
${}^{a}$Note: a values represent mean and the associated standard deviation; p < 0.05 is considered statically significant			
One-way ANOVA and Tukey tests were used to determine significance			
CC: clomiphene citrate HCG: human chorionic gonadotropin			

**Table 3:** Expression levels of endometrial epithelium CD98 in non-ovariectomized subgroups exposed to CC with and without HCG and comparison with control group


**Subgroups (each, n = 6)**	**Non- ovariectomized group**	**Control group (n = 6)**	**p** *${}^{a}$*
CC10 (mg/kg)	1.86 ± 0.22	0.0001
CC20 (mg/kg)	1.22 ± 0.28	0.0001
HCG (7.5IU/kg)	4	1
CC10(mg/kg) + HCG (7.5I.U)	2.69 ± 0.38	0.0001
CC20(mg/kg) + HCG (7.5I.U)	1.80 ± 0.82	3.93 ± 0.35	0.0001
${}^{a}$Note: a values represent mean and the associated standard deviation; p < 0.05 is considered statically significant			
One-way ANOVA and Tukey tests were used to determine significance			
CC: clomiphene citrate HCG: human chorionic gonadotropin			

**Table 4:** Dosage-related effects of CC on expression levels of endometrial epithelium CD98


	**Ovariectomized**				**Non-ovariectomized**			
	CC10 (mg/kg)	CC20 (mg/kg)	CC10 (mg/kg) + HCG	CC20 (mg/kg) + HCG	CC10 (mg/kg)	CC20 (mg/kg)	CC10 (mg/kg) + HCG	CC20 (mg/kg) + HCG
Mean ± SD	1.12 ± 0.30	1	1.78 ± 0.63	1.15 ± 0.211	1.86 ± 0.22	1.22 ± 0.28	2.69 ± 0.38	1.80 ± 0.82
P	0.96		0.002		0.005		0.0001	
Note: A value represent mean and the associated standard deviation; (p < 0.05) is considered statistically significant; a dose-dependent decrease in CD98 was significantly observed with increasing doses of CC from 10 to 20 mg/kg								
One-way ANOVA and Tukey test were used to determine significance								
CC: clomiphene citrate HCG: human chorionic gonadotropin								

**Table 5:** Changes in the expression of endometrial epithelium CD98 due to the administration of HCG following different doses of CC


	**Ovariectomized**				**Non-ovariectomized**			
	CC10 (mg/kg)	CC10 (mg/kg) + HCG	CC 20 (mg/kg)	CC20 (mg/kg) + HCG	CC10 (mg/kg)	CC10 (mg/kg) + HCG	CC20 (mg/kg)	CC20 (mg/kg) + HCG
Mean ± SD	1.12 ± 0.30	1.78 ± 0.63	1	1.15 ± 0.211	1.86 ± 0.22	2.69 ± 0.38	1.22 ± 0.28	1.80 ± 0.82
P	0.0001		0.016		0.001		0.919	
Note: A value represent mean and the associated standard deviation; (p < 0.05) is considered statistically significant		
One-way ANOVA and Tukey test were used to determine significance		
CC: clomiphene citrate HCG: human chorionic gonadotropin		

## 4. Discussion

As our best knowledge this is the first experimental study that shows the expression of CD98 on endometrial epithelium in adult female rats (ovariectomized and non-ovariectomized) after exposure to two doses of CC and HCG alone and combined. Our results revealed a significant decrease in the expression of CD98 after treating with CC alone and in combination with HCG. Also, the adverse effects of CC on the expression levels in ovariectomized rats were more prominent. In addition, the administration of HCG following a lower dose of CC (10 mg/kg) significantly increased the expression of the CD98, but it could not fully reverse it to the normal status. Additionally, the expression of endometrial epithelium CD98 in both ovariectomized and non-ovariectomized showed that a CC dose-dependent manner and low dosage was less destructive. Finding the molecular basis of embryonic implantation is very important clinically and biologically (7). A number of studies have shown that molecular control of endometrial receptivity involves the interface between a diversity of molecules including lectins, mucins, glycoproteins, and other endometrial secretory products (25). One of these molecules is CD98, a member of tetraspanin-enriched microdomains family, which has been shown to be expressed in human endometrial epithelium and appears to be an important aspect of human endometrial receptivity during the implantation window (7).

The results of Domínguez and colleagues are the same as that of Thomas and colleagues that showed that TGFβ (Transforming Growth Factor) and gonadotropins significantly reduced CD98 (in-vitro) and integrins (α1β1, α4β1 and αvβ3) expression, respectively. Also, Meyer and coworkers showed a reduction in the expression of integrins in women who underwent ovulation induction (26) which is consistent with the expression of CD98 in our animals treated with CC, although the experimental design with respect to the case, drug, dosage, and duration of treatments was not similar. The reduction can be explained by a blockage of estradiol 2 [(E2)-induced endometrial epithelial cell proliferation], transcriptional functions of a gene that is responsible for creating different parts of estrogen receptor (ER), formation of ER complex with SRC-1(steroid receptor co-activator-1), and inhibition of mitosis in proliferative phase by CC (27).

However, morphometric evaluation of glandular and stromal structures showed a clear reduction of endometrial development after several treatment protocols (23, 27). The endometrial epithelium may also be affected at molecular levels. Modification in estrogen levels may cause a failure in endometrial receptivity (28). Our results regarding a decrease in CD98 expression of ovariectomized animals treated with CC and HCG alone and in combination are consistent with the results of a previous study by Carpenter and colleagues, who showed that ovariectomy by disturbing the different parts of activin-follistatin system, may negatively affect the growth and development of endometrial glands in ovariectomized animals (29). The ovarian factors that adjust uterine growth and endometrial adenogenesis are not clearly defined, although there are evidences that describe follistatin and inhibin or activin as involved factors (30).

In addition, the maximum expression level in Ovariectomized subgroups belonged to the HCG subgroup, but this amount was significantly less than the normal groups. Thus, the results of this study indicated that a single dose of HCG is unable to repair the adverse effects on CD98 expression due to the lack of steroidal hormones of the ovary and their role in endometrial proliferation. Our results suggest that normal ovaries are necessary for normal expression of CD98; however, additional studies are needed to clarify the effects of CC and HCG alone and combination of following ovariectomy on the expression of CD98. It has been suggested that CC may increase serum levels of estradiol and progesterone and decrease endometrial epithelium there upon (4) and a higher CC dosage usually resulted in great diverging morphological alterations. We found that the expression of CD98 was related to CC dosage when used alone in Ovariectomized subgroups and with HCG in both Ovariectomized and non-Ovariectomized subgroups; this confirms the results of a previous studies that declared a dose-related effect of the CC on endometrial structure and steroid concentrations (31). Also, our findings were similar to the results of a study that showed that increased doses of CC from 1, 5 to 10 µ M in the culture medium of endometrial cells interrupted natural proliferation and modification of cells (32).

The conflicting results of CC may be due to its antiestrogenic effects on endometrium as well as cervix. In our study, HCG failed to reverse the expression levels to normal values following pre-treatment with CC; also, the data revealed that a single dose of HCG alone markedly increased the expression of CD98. In addition, HCG significantly affects the expression of the CD98 in the both experimental subgroups that received a lower dose of CC (10mg/kg). These results are in agreement with a report by Dominguez and colleagues, who detected that the greatest increase observed upon the treatment with both progesterone and 17-β-estradiol, and HCG treatment is also able to induce the expression of CD98 in primary endometrial epithelial cells (7). A possible explanation for this could be that during the window of implantation, HCG exerts a positive influence on endometrial epithelium to secret several growth factors (interleukin-11, CXCL10, granulocyte macrophage colony-stimulating factor) that resulted in more proliferation of endometrium, increase in reception and development of trophoblast (33). Also, it may particularly induce decidualization by rising cAMP synthesis (34). In addition, it has been suggested that multiple paracrine factors (as tissue remodeling parameters) related to the endometrial differentiation include; insulin-like growth factor-binding protein-1(IGFBP-1), vascular endothelial growth factor (VEGF), and matrix metalloproteinase-9 (MMP-9) can be modulated by HCG administration during the luteal phase (30). Additionally, it is confirmed that HCG can modulate the secretion of cytokines and growth factors thorough LH receptors and improve endometrial epithelium and implantation (20, 35).

## 5. Conclusion

Our results indicated that CC impairs CD98 expression in endometrium and this impairment is intensified with the removal of the ovary. Also, it appears that HCG has slightly stimulatory responses on CD98 gene expression, thus it can slightly elevate its expression depending on the CC dose. In addition, the dose 20 mg/kg has the most negative effects on the gene expression of CD98. More studies are recommended in this field to investigate other molecular and cellular mechanisms altered by ovary inductive treatments.

##  Conflict of Interest

The authors declare that they have no conflict of interests.

## References

[1] Agrawal K, Gainder S, Dhaliwal LK, Suri V. Ovulation induction using clomiphene citrate using stair-step regimen versus traditional regimen in polycystic ovary syndrome women- a randomized control trial. *J Hum Reprod Sci* 2017; 10: 261–264. 10.4103/jhrs.JHRS_15_17 PMC5799929 29430152

[2] Selim MF, Tamer Farouk B. Letrozole and clomiphene citrate effect on endometrial and subendometrial vascularity in treating infertility in women with polycystic ovary syndrome. *J Gynecol Surg* 2012; 28, 33–39.

[3] Nelson LM, Hershlag A, Kurl RS, Hall JL, Stillman RJ. Clomiphene citrate directly impairs endometrial receptivity in the mouse. *Fertil Steril* 1990; 53: 727–731. 2318331

[4] Lacin S, Vatansever S, Kuscu NK, Koyuncu F, Ozbilgin K, Ceylan E. Clomiphene citrate does not affect the secretion of alpha3, alphaV and beta1 integrin molecules during the implantation window in patients with unexplained infertility. *Hum Reprod* 2001; 16: 2305–2309. 10.1093/humrep/16.11.2305 11679509

[5] Tassell R, Chamley WA, Kennedy JP. Gonadotrophin levels and ovarian development in the neonatal ewe lamb. *Aust J Biol Sci* 1978; 31: 267–273. 10.1071/bi9780267 727995

[6] Klein M. The impact of accounts and attributions following marital infidety. Columbia: University of Missouri; 2007: 1–147.

[7] Dominguez F, Simon C, Quinonero A, Ramirez MA, Gonzalez-Munoz E, Burghardt H, *et al* Human endometrial CD98 is essential for blastocyst adhesion. *PloS One* 2010; 5: e13380. 10.1371/journal.pone.0013380 PMC2955532 20976164

[8] BildacıTB, Haydardedeoğlu B, Karakaya BK, Bolat FA, Zeyneloğlu HB. The importance of CD56 and CD98 levels in patients with recurrent implantation failure. *J Clin Anal Med* 2017; 8: 216–218.

[9] Dazzi L, Seu E, Cherchi G, Barbieri PP, Matzeu A, Biggio G. Estrous cycle-dependent changes in basal and ethanol-induced activity of cortical dopaminergic neurons in the rat. *Neuropsychopharmacology* 2007; 32: 892–901. 10.1038/sj.npp.1301150 16841076

[10] Lessey BA, Damjanovich L, Coutifaris C, Castelbaum A, Albelda SM, Buck CA. Integrin adhesion molecules in the human endometrium. Correlation with the normal and abnormal menstrual cycle. *J Clin Invest* 1992; 90: 188–195. 10.1172/JCI115835 PMC443080 1378853

[11] Hosie M, Adamson M, Penny C. Actin binding protein expression is altered in uterine luminal epithelium by clomiphene citrate, a synthetic estrogen receptor modulator. *Theriogenology* 2008; 69: 700–713. 10.1016/j.theriogenology.2007.12.002 18258291

[12] Xu X, Liu ML, Lu J, Xie P, Song HP. [Effects of long-term estrogen replacement treatment on the expression of bcl-2 and H-ras in rat endometrium]. *Zhong Nan Da Xue Xue Bao Yi Xue Ban* 2005; 30: 41–45. (in Chinese) 15871186

[13] Salgado RM, Favaro RR, Zorn TM. Modulation of small leucine-rich proteoglycans (SLRPs) expression in the mouse uterus by estradiol and progesterone. *Reprod Biol Endocrinol* 2011; 9: 22–29. 10.1186/1477-7827-9-22 PMC3041739 21294898

[14] Ciortea R, Costin N, Braicu I, Haragas D, Hudacsko A, Bondor C, *et al* Effect of melatonin on intra-abdominal fat in correlation with endometrial proliferation in ovariectomized rats. *Anticancer Res* 2011; 31: 2637–2643. 21778316

[15] Casper RF. It's time to pay attention to the endometrium. *Fertil Steril* 2011; 96: 519–521. 10.1016/j.fertnstert.2011.07.1096 21880272

[16] Zhao J, Zhang Q, Li Y. The effect of endometrial thickness and pattern measured by ultrasonography on pregnancy outcomes during IVF-ET cycles. *Reprod Biol Endocrinol* 2012; 10: 100–111. 10.1186/1477-7827-10-100 PMC3551825 23190428

[17] Fenczik CA, Sethi T, Ramos JW, Hughes PE, Ginsberg MH. Complementation of dominant suppression implicates CD98 in integrin activation. *Nature* 1997; 390: 81–85. 10.1038/36349 9363894

[18] Cantor JM, Ginsberg MH. CD98 at the crossroads of adaptive immunity and cancer. *J Cell Sci* 2012; 125: 1373–1382. 10.1242/jcs.096040 PMC3336374 22499670

[19] Radesic B, Tremellen K. Oocyte maturation employing a GnRH agonist in combination with low-dose hCG luteal rescue minimizes the severity of ovarian hyperstimulation syndrome while maintaining excellent pregnancy rates. *Hum Reprod* 2011; 26: 3437–3442. 10.1093/humrep/der333 21997895

[20] Rao CV. Physiological and pathological relevance of human uterine LH/hCG receptors. *J Soc Gynecol Invest* 2006; 13: 77–78. 10.1016/j.jsgi.2005.12.005 16443498

[21] Tsumura H, Suzuki N, Saito H, Kawano M, Otake S, Kozuka Y, *et al* The targeted disruption of the CD98 gene results in embryonic lethality. *Biochem Biophys Res Commun* 2003; 308: 847–851. 10.1016/s0006-291x(03)01473-6 12927796

[22] Verrey F, Meier C, Rossier G, Kuhn LC. Glycoprotein-associated amino acid exchangers: broadening the range of transport specificity. *Pflugers Arch* 2000; 440: 503–512. 10.1007/s004240000274 10958334

[23] Damario MA, Lesnick TG, Lessey BA, Kowalik A, Mandelin E, Seppala M, *et al* Endometrial markers of uterine receptivity utilizing the donor oocyte model. *Hum Reprod *2001; 16: 1893–1899. 10.1093/humrep/16.9.1893 11527894

[24] Rinaldi L, Lisi F, Floccari A, Lisi R, Pepe G, Fishel S. Endometrial thickness as a predictor of pregnancy after in-vitro fertilization but not after intracytoplasmic sperm injection. *Hum Reprod* 1996; 11: 1538–1541. 10.1093/oxfordjournals.humrep.a019434 8671501

[25] Cai S, Bulus N, Fonseca-Siesser PM, Chen D, Hanks SK, Pozzi A, *et al* CD98 modulates integrin beta1 function in polarized epithelial cells. *J Cell Sci *2005; 118: 889–899. 10.1242/jcs.01674 15713750

[26] Kyrou D, Fatemi HM, Blockeel C, Stoop D, Albuarki H, Verheyen G, *et al* Transfer of cryopreserved - thawed embryos in hCG induced natural or clomiphene citrate cycles yields similar live birth rates in normo-ovulatory women. *J Assist Reprod Genet* 2010; 27: 683–689. 10.1007/s10815-010-9464-x PMC2997952 20703796

[27] Amita M, Takahashi T, Tsutsumi S, Ohta T, Takata K, Henmi N, *et al* Molecular mechanism of the inhibition of estradiol-induced endometrial epithelial cell proliferation by clomiphene citrate. *Endocrinology* 2010; 151: 394–405. 10.1210/en.2009-0721 19934375

[28] Jeschke U, Wang X, Briese V, Friese K, Stahn R. Glycodelin and amniotic fluid transferrin as inhibitors of E-selectin-mediated cell adhesion. *Histochem Cell Biol* 2003; 119: 345–354. 10.1007/s00418-003-0529-0 12743827

[29] Carpenter KD, Hayashi K, Spencer TE. Ovarian regulation of endometrial gland morphogenesis and activin-follistatin system in the neonatal ovine uterus. *Biol Reprod* 2003; 69: 851–860. 10.1095/biolreprod.103.016337 12748121

[30] Wang Y, Chen Y, Li M. [Effects of ovulation induction on expression of integrins alpha 4 beta 1 in endometrium]. *Zhonghua Fu Chan Ke Za Zhi* 2001; 36: 352–354. (in Chinese) 11783134

[31] Dickey RP, Olar TT, Curole DN, Taylor SN, Rye PH. Endometrial pattern and thickness associated with pregnancy outcome after assisted reproduction technologies. *Hum Reprod* 1992; 7: 418–421. 10.1093/oxfordjournals.humrep.a137661 1587952

[32] Khazaei M, Chobsaz F, Khazaei S. [The effect of different doses of clomiphen citrate on morphology and proliferation of human endometrial stromal cells in in-vitro culture.] *J Babol Univ Med Sci* 2010; 12: 7–12. (in Persian)

[33] Paiva P, Hannan NJ, Hincks C, Meehan KL, Pruysers E, Dimitriadis E, *et al* Human chorionic gonadotrophin regulates FGF2 and other cytokines produced by human endometrial epithelial cells, providing a mechanism for enhancing endometrial receptivity. *Hum Reprod* 2011; 26: 1153–1162. 10.1093/humrep/der027 21345913

[34] Tang B, Gurpide E. Direct effect of gonadotropins on decidualization of human endometrial stroma cells. *J Steroid Biochem Mol Biol* 1993; 47: 115–121. 10.1016/0960-0760(93)90064-4 8274425

[35] Tesarik J, Hazout A, Mendoza C. Luteinizing hormone affects uterine receptivity independently of ovarian function. *Reprod Biomed Online* 2003; 7: 59–64. 10.1016/s1472-6483(10)61729-4 12930575

